# Specialized Metabolites and Valuable Molecules in Crop and Medicinal Plants: The Evolution of Their Use and Strategies for Their Production

**DOI:** 10.3390/genes12060936

**Published:** 2021-06-18

**Authors:** Vincenzo D’Amelia, Teresa Docimo, Christoph Crocoll, Maria Manuela Rigano

**Affiliations:** 1 Institute of Bioscience and BioResources, National Research Council, Via Università 100, 80055 Portici, NA, Italy; 2 DynaMo Center, Department for Plant and Environmental Sciences, University of Copenhagen, Thorvaldsensvej 40, 1871 Frederiksberg, Denmark; chcr@plen.ku.dk; 3 Department of Agricultural Science, University of Naples Federico II, Via Università 100, 80055 Portici, NA, Italy

**Keywords:** bioactive compounds, genomics, metabolomics, genome editing, medicinal plants, plant natural products, plant biotechnology, biofortification

## Abstract

Plants naturally produce a terrific diversity of molecules, which we exploit for promoting our overall well-being. Plants are also green factories. Indeed, they may be exploited to biosynthesize bioactive molecules, proteins, carbohydrates and biopolymers for sustainable and large-scale production. These molecules are easily converted into commodities such as pharmaceuticals, antioxidants, food, feed and biofuels for multiple industrial processes. Novel plant biotechnological, genetics and metabolic insights ensure and increase the applicability of plant-derived compounds in several industrial sectors. In particular, synergy between disciplines, including apparently distant ones such as plant physiology, pharmacology, ‘omics sciences, bioinformatics and nanotechnology paves the path to novel applications of the so-called molecular farming. We present an overview of the novel studies recently published regarding these issues in the hope to have brought out all the interesting aspects of these published studies.

## 1. Valuable Molecules from Plants: An Ancient Resource for Modern Challenges

The world is facing several environmental, economic and social challenges. Climate change, soil desertification, the ageing population, pandemics, pollution and the severe depletion of natural resources are steering public research and the industry to look for innovative solutions to grant health and safety for the future. The common research policy addresses towards the preservation of natural resources by promoting the use of renewable materials and sustainable industrial processes. Plants represent an important solution in this context. For example, plant-derived products are used to improve food quality with healthy ingredients, and they can be used in form of dietary supplements to contribute to a healthy diet by providing vitamins, antioxidants and fibres. It has been proven that the daily consumption of biofortified foods and beverages with healthy ingredients can help prevent several non-communicable diseases such as cardiovascular diseases, cancer, neurodegenerative diseases and diabetes [1]. The recognition of the importance of food in healthcare, whose benefit goes beyond its nutritional value, has led the global market value of the so-called “functional foods” to reach USD 300 billion in 2020 as estimated by Guiné et al., 2020 [2]. Regional regulatory bodies stimulate the development of research projects aiming to identify new phytochemicals, also from underutilized plants, that can be exploited to design new nutraceuticals or functional foods [3]

The pharmaceutical sector is undoubtedly largely inspired by plant-derived compounds. In the second half of the twentieth century, the interest in plant specialized metabolites increased in Western medicine, especially for those molecules, such as atropine, codeine, digoxin, morphine, quinine, placlitaxel and vincristine, which actually remain elective drugs for the treatment of several illnesses. More than 70% of the 1562 approved drugs from natural origins have been introduced between 1981 and 2014 [4] and about 90% of the medicinal plants used in Europe are harvested from wild resources [5]. More recently, Newman and Cragg, 2020 [6], reported that in the last forty years, 929 out of 1881 new approved drugs have a natural origin, and another 952 drugs, although classified as synthetic drugs, either possess natural pharmacophores or molecules mimicking a natural product. Market prediction indicates that the global market for botanical and plant-derived drugs will grow from USD 29.4 billion in 2017 to around USD 39.6 billion by 2022, with a compound annual growth rate (CAGR) of 6.1% during the period 2017–2022 [7].

Nevertheless, the increased demand for bioactive molecules derived from plants has an ecological risk. It may lead to an over-exploitation of plant resources, ultimately causing biodiversity loss [8]. Several solutions can be pursued to avoid this dramatic drawback. In situ and ex situ conservation, the domestication of wild species, preservation through botanical garden and bank seeds are necessary priorities for biodiversity protection. We found the solution proposed by some authors to cultivate orphan crops or wild relatives of cultivated crops (defined as third generation crop) to directly replace lignocellulosic and biomass species (e.g., *Mischantus*, poplar, willow) particularly attractive. For these latter species, there are concerns about their impact on biodiversity, as these are increasingly cultivated in extensive monoculture [9,10]. The above proposed solution may provide the opportunity to re-introduce several wild species, which are more climate resilient than crops, and that can also be exploited for other human needs. For example, foxtail millet (*Setaria italica*) has been evaluated as particularly apt to produce biofuel, but it can also provide beneficial phytosterols that can be used as dietary supplements [11,12,13].

The over-exploitation of plant resources can also be prevented thanks to the fact that in the last ten years, the emergence of high-throughput techniques in all the ‘omics’ sciences allowed to reduce the number of samples and the time necessary for massive screening analyses. Sequencing and transcriptomic data added novel knowledge on genes and enzymes responsible for specific metabolite biosynthesis, thus supporting plant selection, marker assisted breeding for quality traits and even the exploitation of heterologous systems for producing target molecules. Advances in chemical analytical methodologies in mass spectrometry and NMR coupled with progress in tissue and single cell isolation are facilitating the massive biological characterization of plant extracts, especially for drugs which are often accumulated in low amount in plant tissues. These systems allow a lower amount of plant materials to be screened and an increased sensitivity in molecule detection, giving us the possibility of using a more nature-based approach able to preserve biodiversity [14,15]. Simultaneously, the development of genetic engineering strategies and micropropagation techniques are also effective alternatives in preserving these resources, and scientific research on plant-derived drugs are particularly focused on the use of these biotechnological approaches [16].

Bearing in mind these concepts, we dissected the main outcomes as well as all the aspects dealing with the genetic and most innovative biotechnological strategies to exploit plant-derived compounds. In particular, we discussed the studies published in the Special Issue “Genetic and Molecular Mechanisms in Plant-Derived Compounds for Industrial Purposes” of the *Genes* journal (https://www.mdpi.com/journal/genes/special_issues/plant_derived_compounds), in the context of existing knowledge.

## 2. Molecules in Crop and Medicinal Plants: The Evolution of Their Use

Phytochemicals were used in traditional human medicine since long before recorded history. They were used as pigments for handcrafts and cosmetics, scents during rituals and terrible poisons that marked mysterious historical events. Even the preliminary attempts to systematically group plants were based on traits determined by phytochemicals. Before the revolutionary works of Linneaus (i.e., Systema Naturae, 1735 and Species Plantarum, 1753) traits like savory, spiciness, along with medical and artisanal uses, suggested people and even naturalists how to group herbals (examples are in work *De Materia Medica* of Dioscorides, 1st century A.D. and *Theatrum Botanicum* of Parkinson, 1640). This anthropocentric thought is well expressed in Paraceulsus’ belief (1493–1541) that God indicated to humans what the right healing applications of the plant substances were through plant organ morphology. Following this idea, for example, the St. John’s wort (*Hypericum perforatum* L.) would be beneficial for the treatment of wounds, since the leaves appear as if they had been stung [17]. Indeed, the translucent glands of St John’s wort, that are concentrated in the leaf lamina, make the leaves appear perforated when observed under blacklight [18]. Though we can state that the idea of Paracelsus was far from reality, ironically, these translucent glands, along with others named black glands, represent the major secretory structures of the bioactive compounds produced by St John’s wort. The modern biochemical characterization of the Hypericum extracts offered the possibility of novel pharmaceutical applications, which are beyond the traditional wound healing ones. As a matter of fact, the two main bioactive molecules produced by Hypericum, hyperforin and hypericin, showed antidepressant and anti-cancer properties and could be a potential drug for neurodegenerative diseases [18]. Generally, the uses of medicinal plants vary according to their cultural context. In addition, the evolution of the research methodology reveals the novel beneficial properties of plant extracts. Leonti and Verpoorte, 2017 [19], discussed the needs for developing an ethnopharmacological database that collects data on both historical and modern uses of medical herbs. We found that the NAPRALERT^®^ database (https://www.napralert.org), IMPPAT^®^ (http://cb.imsc.res.in/imppat/home) or TCMID ^®^ (http://bidd.group/TCMID/) were particularly interesting, since these include data from the literature on natural products, pharmacological/biochemical bioactivities and ethnomedical information. Databases displaying a more molecular tendency are, for example, the Herbal Medicine Omics Database (HMOD) and the Plant Metabolic Network (PMN), that integrate metabolic and biochemical data with publicly available genomic, transcriptomics and enzymatic databases [20,21]. These data may serve as the basis for in silico processing and to computationally reconstruct a single specific pathway, and thus assess the broader roles of the structural genes involved in the pathway of interest and to facilitate the discovery of new enzymes. For example, Chae et al., 2014 [22] published a very interesting work where they described a set of the different genomic signature of genes encoding for enzymes of specialized metabolism. The authors predicted the use of these genomic data as a tool for the rational discovery of genes involved in novel specialized molecules in different plants. Similarly, Schlapfer et al., 2017 [23], predicted the metabolic pathways for 21 plant and one algal species, including major crops and model organisms, by using the PMN database. In this latter work, the authors gave particular emphasis to gene clusters. Gene clusters are physically linked clusters of genes that encode for signature and tailored enzymes involved in the definition of a specific compound, whose identification may help finding new pathways and new specialized metabolites [24]. The outcomes of these studies may also provide useful materials to design valuable strategies to produce tailored compounds by using synthetic biology approaches. Examples of synthetic biology approaches will be discussed in a later section.

The knowledge on the different uses of a medicinal plant all over the world opens new horizons for scientific research that allows these species to be valorised. For example, *Dalbergia odorifera* is not only a source of precious rosewood, but an important medicinal plant in Asian folk pharmacopoeia. Sun et al., 2020 [25], reported the double properties of flavonoids and sesquiterpenes from *D. odorifera*, which contribute to the peculiar heartwood scent and colours of rosewood, as well as represent bioactive molecules beneficial for human health. Bioactive molecules easily become commodities, transforming noxious weeds into valuable crops. The milk thistle (*Silybum marianum*) is generally used for bioenergy production and for phytoremediation. However, the main molecules produced, silymarin or silybin, have generated considerable interest in cosmetic manufacturing [26], and recently, milk thistle has been found to hold anti-genotoxic compounds that promote the use of its extract as an anti-ageing product [27]. Similarly, among Asteraceae, a sister species of milk thistle, cardoon (*Cynara cardunculus* var. altilis), which historically played a limited role as a traditional food and as a vegetable rennet substitute for cheese making [28], has attracted a lot of research interest in the last thirty years for its wide spectrum of different uses. Indeed, cardoon tissues and organs are a rich source of oils and bioactive compounds providing formidable antioxidants [29], and its vigorous biomass growth even under hostile environmental conditions [30], in addition to its seed oil content and composition, are valuable for energy production [31].

Today, crop biofortification projects are exponentially increasing. They represent a favourable strategy in view of drastic climate change, which is already causing the decline of available dietary micronutrients and thus further compromising health in populations with limited or no access to alternative sources of micronutrients, supplements, or more diverse diets [32]. Generally, most the relevant research programs have been developed to provide seven micronutrient-rich crops, namely iron beans, iron pearl millet, vitamin A cassava, vitamin A maize, vitamin A sweet potato, zinc rice, and zinc wheat [33]. An example is the HarvestPlus, an international research program (http://www.harvestplus.org) whose main scope is the biofortification of several stable crops with micronutrients. The European commission also supports the crop biofortification proposal. For example, Bio-BELIEF (https://www.era-learn.eu/network-information/networks/fosc/food-systems-and-climate-call-2019/biofortification-of-common-bean-to-promote-healthy-diet-and-food-security-in-a-context-of-climatic-variation), OR4FOOD (https://or4food.cirad.fr/en/project) and Se4All (https://cordis.europa.eu/project/id/101007630) are only a few examples of currently financed research projects that address micronutrient biofortification in plant-derived products through the use of both conventional agricultural/breeding strategies and modern biotechnological approaches.

Genetic studies combined with food technological research are more and more common for crop species, with the aim of identifying genomic regions that can serve as tools in breeding programs to adapt crop-derived compounds to the physical–chemical conditions of food technological processes [34,35]. There is increasing interest in the release of novel crop varieties coloured with purple or blue pigments and novel studies are testing the beneficial properties of these pigments against several human pathologies [34,36]. Flavonoids, such as flavonols and anthocyanins, are an example of crop-derived compounds largely subjected to both industrial and medicinal applied studies, and these are drawing increasing interest because of their discovered bioactivity against COVID-19 [37]. It is worth mentioning the comprehensive review by Iorizzo et al., 2020 [38], regarding the use of the flavonoid anthocyanins from carrots as powerful natural food pigments. The authors also discussed the use of anthocyanin decorative genes, i.e., genes involved in the polyglycosylation, methylation and acylation of anthocyanins, as an important tool to extend anthocyanin profiles, and consequently, their chemical properties, including bioavailability and stability, important for medicinal and industrial purposes. The presence of patents related to the use and production of anthocyanins has also been discussed by Iorizzo and colleagues, 2020 [38], further highlighting the real applicability of these studies in the industrial sector. We took a quick electronic survey on the patents released in the last 20 years regarding the use of biotechnological methods to improve the production of plant-derived compounds. We report in Table 1 an example of these methods, including the use of either improved media and elicitors or the use of single gene transgenic approaches to boost the production of metabolites in cell cultures as well as the use of a synthetic biology method to transfer optimized plant genes in the heterologous system. Moreover, we cited the patent num. US8158857B2 as an example of the use of plants as biofactories for the production of exogenous peptides. These methods will be further described in the next paragraphs.

## 3. Production Improvement of Plant-Derived Compounds by Stress Elicitation

Quite severe weather changes have occurred in the last ten years and the impacts of climate change appear to deeply affect crop productivity. Several studies [25,49,50,51,52] documented the effect of unexpected heat, cold, light, drought and salinity stresses on plant metabolism and growth, which often is severely impaired [53,54,55,56,57]. The evaluation of the impact of changing environmental factors on plants, as well as the knowledge of plant responses, is helpful to design a potential strategy to minimize this impact by selecting species and varieties with favourable traits able to confer adaptation and even resistance.

**Figure 1:**
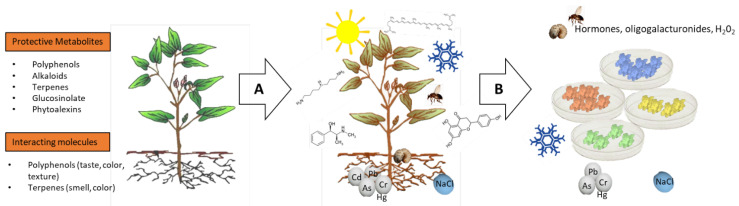
(**A**) Plants produce several bioactive molecules that have a protective or interacting role in the surrounding environment. (**B**) The biosynthesis of these metabolites is boosted during environmental cues, as well as biotic and abiotic stresses. (**C**) The environmental constraints or the plant endogenous signalling molecules, which induce the compound accumulation, can be used as an “elicitor” to boost the production of molecules with industrial interest, including in in vitro culture systems (e.g., undifferentiated cell cultures).

Plants produce a wide arsenal of specialized metabolites, a shield against changing constraints from the environment [58,59,60]. Specialized metabolites, differently from constitutive primary metabolites, are often restricted to specific plant species and produced upon environmental stimulation. The peculiar production of specialized metabolites, although energetically costly for the plant, is beneficial for the interaction with the environment, and in some cases, necessary for survival in a hostile environment. The fascinating complexity of these compounds reflects the plant capability to communicate with the surrounding ecosystem. The large number of different molecules that can be produced by plants also mirrors the extensive number of environmental and ecological changes that plants might have experienced during their evolution. In this regard, the fast global environmental changes, such as climate warming, extensive deforestation, as well as air, water and soil contamination are compromising the stability of the system upon which all organisms depend. All these factors determine the ecological challenges for the survival of the local biological communities, including plants, animals and microbes, as well as positive opportunities for invasive exotic species. These environmental and ecological changes determine novel plant–environment interactions [61], often mediated by novel metabolic capabilities. The highly complex and evolute molecular regulation of these metabolites grants plants a prompt and specific reaction towards external stimuli and against stressors [58,62,63]. For example, interactive specialized metabolites confer colour, taste, odour and floral scents to plants, and these characteristic plant signals allow plants to communicate attraction or repellence to other plants and living organisms (Figure 1). In addition, since they are sessile organisms and through the accumulation of specific bioactive compounds, plants have learnt how to protect themselves from abiotic stresses such as high UV light, drought, high and low temperatures and biotic stresses caused by other living organisms such as parasites and pathogens (virus, bacteria, fungi, nematodes or insects) (Figure 1) [64,65,66,67,68]. When plants face environmental stresses, these produce reactive oxygen species (ROS) that work as signalling molecules and activate defence-related genes along with a sophisticated biochemical response cascade through phytohormones [69]. At the same time, plants need to be protected by the oxidative burst, since ROS can strongly damage vital macromolecules such as DNA, proteins and lipids. When the antioxidant enzymes machinery results insufficient, polyphenols, above all specialized molecules, become a strong tool in buffering ROS accumulation and flavonoid class, in particular, represents an important defence against abiotic stress [70]. The interaction between plant and pathogen produces a similar route of response. ROS signalling through mitogen-activated protein kinases (MAPKs) results in local or systemic defence responses [71]. The attack of pathogens on the cell wall or also simple wounding determines the release of peroxidases into the apoplast, resulting in an oxidative burst [72]. Through fine-tuning, peroxidases contribute to either ROS formation or degradation. Under pathogen attack, ROS formation facilitates the degradation of pathogen proteins and nucleic acids, thus directly killing the pathogen [72]. The intricate molecular mechanism through which plants can adapt to the environment is not yet fully explored, however, well-known signals such as the oligogalacturonides, jasmonic acid, hydrogen peroxide, ABA and salicylic acid signalling pathways are commonly used for plant “elicitation” to stimulate stress responses and boost the production of molecules for industrial purposes (Figure 1).

Specialized metabolites, due to their reactive nature, are often produced in very low amounts and in specific ways in different species or genus. In addition, their biosynthesis might only be activated during a particular growth or developmental stage, or under specific conditions related to the season, stress, or nutrient availability. Researchers are making remarkable advances in understanding plant-derived compounds’ biosynthesis and regulation with the aim of better manipulating metabolite production [73], especially by using in vitro growth of plant cells, tissues and organ cultures under a controlled environment.

### 3.1. Laboratory Scale

In the Special Issue “Genetic and Molecular Mechanisms in Plant-Derived Compounds for Industrial Purposes” of the *Genes* journal (https://www.mdpi.com/journal/genes/special_issues/plant_derived_compounds), several manuscripts [18,40,41,42,43] describe the use of abiotic stresses such as wounding, nutrient starvation, temperature and light stresses as strategies for improving the production of bioactive compounds. For example, the work by Sun et al., 2020 [25], describes the development of a method to increase the production of specific flavonoid compounds in *Dalbergia odorifera*. This species represents the most precious rosewood in China, as it is a very slow growing small tree; indeed, the development of valuable heartwood requires almost 50 years under natural conditions. The heartwood is not only used for the production of precious woods furniture, but also as a source of bioactive molecules upon wounding. The authors, aware of the importance of biodiversity conservation and the preservation of endangered species, studied the effects of wounding stress in *D. odorifera* with the final aim of developing a method for the production of elicited suspension cultures and to provide a more sustainable approach to recover valuable metabolites. Using RNA sequencing, Sun et al., 2020 [25] demonstrated that ethylene, jasmonic acid, hydrogen peroxide, salicylic acid and abscisic acid mediate wounding-induced flavonoid production and suggested that researchers could use these compounds as elicitors of the flavonoid pathway.

The maintenance of a stable accumulation of molecules is an important prerequisite for commercially crops, especially for those crops consumed as food or used in beverages. Su et al., 2020 [51], highlighted the impact of nutrient starvation on tea quality as mainly due to the number of specific compounds accumulated in the leaves of the tea plant (*Camellia sinensis*) during its cultivation. The single effect of N, P and K nutrient starvation on the biosynthesis of key flavour determinants of tea was investigated by RNA sequencing. Comparative reports indicated that each micronutrient was able to exert a specific effect on primary and secondary metabolism, without excluding the existence of common molecular networks in response to these macronutrient stresses. Interestingly, N starvation was mostly effective on catechins accumulation, whereas P and K starvation determined a marked reduction in caffeine and K starvation, as well as of L-theanine accumulation. Moreover, the integration of metabolic and transcriptomic data led to the identification of 35 transcription factors (TFs), belonging to the MYB, WRKY, bHLH, and ERF TF families, highly correlated with total catechin changes.

Anthocyanins have attracted significant interest from the scientific community for their versatile uses. Since the induction of anthocyanins is easily detectable to the naked eye, several elicitors can easily be screened to increase the accumulation of these high-value natural molecules. Several abiotic stresses have been reported to elicit anthocyanin metabolism [74,75,76]. Findings from He et al., 2020 [77,78] demonstrated that low temperatures affect anthocyanin biosynthesis in Chinese cabbage seedlings by promoting a high expression of the positive regulatory genes *Brmyb2* and *Brtt8*, and, thus, determine the cabbage’s purple pigmentation. Interestingly, this study highlighted that at room temperature, the early part of the phenylpropanoid metabolic pathway occurs in all Chinese cabbages, and that the formation of ubiquitous phenylpropanoids is mostly part of the normal physiological metabolism. On the contrary, the production of anthocyanins is slightly repressed at room temperature but promptly induced by cold stress [77,78].

Similarly to cold temperature, light is one of the key environmental factors that stimulates anthocyanin biosynthesis and accumulation in plants [79,80,81]. Light transduction signalling is particularly subjected to post-transcriptional and post-translational regulation, which can impair the direct relationship between transcript and metabolites. The work by Hong et al., 2019 [50], attempted to solve this gap and investigated the light-mediated process of colour formation in chrysanthemum (chrysanthemum × morifolium) by combining transcriptomic and proteomic data. This study proposed that the complementation of RNA sequencing data with iTRAQ data is an efficient method for profiling the candidate proteins involved in physiological processes, rather than the use of a single methodology. In particular, light-induced and downregulated genes were compared with the related differentially expressed proteins quantified by isobaric tags (iTRAQ). The authors confirmed the key interaction between COP1 (constitutively photomorphogenic 1) and HY5 (long hypocotyl 5) in mediating the signalling cascades deputed to the photomorphogenic changes occurring in plants under dark and light conditions [82,83,84]. They reported the activation of anthocyanin negative regulators under dark conditions (e.g., MYB4 and MYB5) and the induction of the genes coding for HY5, MYB6 and MYB7 under light conditions, which were considered putative positive regulators of anthocyanin structural genes. Finally, Hong et al., 2019 [52], proposed another study where they confirmed the role of the above-identified regulators through their functionalization in tobacco. Altogether, the transcriptomic and proteomic analyses may represent a more reliable instrument to select candidate genes for developing genetic markers for breeding new chrysanthemum cultivars resistant to low-light conditions and with possible application in the metabolic engineering of flower colours.

The above-reported studies refer to experimental approaches aiming to understand the inducible nature of plant bioactive compounds and the regulatory mechanisms behind their production, rather than provide an immediate practical application for increasing metabolites. However, we are aware that the use of eliciting molecules in agriculture, to guarantee crop protection and pest management, is a difficult task. In fact, the cost of elicitors is often expensive (e.g., about EUR 130/100 mg-1 for jasmonic acid) and may not find a convenient application in open field systems. The eliciting approaches in laboratory experimental practice still allow us to smartly study the molecular network underlining plant response to pathogen attack or, in general, to environmental constraints. In the future, data coming from these studies may be translated to indicate more sustainable agronomic practices. Moreover, these data are extremely useful to design further biotechnological strategies for industrial scale production of plant-derived metabolites in plant cell cultures or using synthetic biology approaches.

### 3.2. Industrial Scale

Plants are the main source of phytochemicals used for a wide range of applications including pharmaceuticals, agrochemicals, fragrances, biopesticides, and food additives. Considering the need to achieve sustainable growth, a reduction in global warming, and an increase in food supply, plant-derived compounds, especially for industrial purposes, need to be produced in alternative sources to plants. In this context, plant cell culture technology has the potential to provide a sustainable supply of phyto-ingredients with less global energy expense. This is particularly true for bioactive compounds, which are produced in plants with a slow growth rate, or produced in a very tiny amount, or even produced in a wild plant which cannot be cultivated on an agricultural scale [85]. Moreover, plant cell cultures offer the advantages of ensuring a stable supply of plant-derived compounds independently from seasonal and geographical variations, with a sustainable production, a high rate of metabolite production, and according to good manufacturing practices (GMP) [86]. The most important advantage in the use of plant cell cultures is that the biosynthetic pathway for a complex metabolite is already present in the cell and there is no need for it to be re-constructed by using synthetic biology approaches. Indeed, the employment of plant cell culture at the industrial scale is continuing to increase, especially for pharmaceuticals and food supplements [87,88,89]. Examples of the actual use of phytochemicals as pharmaceuticals, food additives and cosmetic ingredients, produced trough plant cell cultures, are reported in Table 2.

However, plant cell culture can encounter several obstacles such as low yields, performance instability and slow plant cell growth. The use of elicitors for increasing the production of a specific valuable compound is often affordable for biotechnological industries (Table 3 and references therein); nevertheless, it might suffer from several limitations. In Table 3, we report the most promising elicitation approaches employing biotic and abiotic elicitors for the sustainable production of plant secondary metabolites of commercial interest.

We wondered which commercial product is produced through the use of the elicitation strategies we discussed above. At the bioreactor scale, preliminary tests are necessary to select suitable elicitor(s) and to determine the optimum addition time, dosage and exposure time of the elicitor. This preliminary process might ultimately be very complex and time consuming [104]. Moreover, for high-value pharmaceuticals, the use of elicitors must meet safety requirements, whereas for the production of food additives, several aspects such as safety and flavour aspects, and ultimately but most importantly, food regulation compliance, need to be carefully observed [105]. However, it was particularly tricky to have this information, most probably because these are not publicly available and/or protected by industrial secret.

## 4. Plant as Biofactories

**Figure 2:**
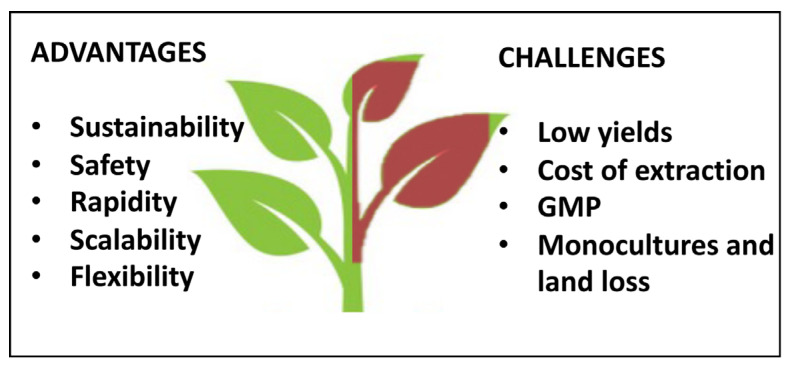
Advantages and disadvantages of plant molecular farming.

Thus far, in this review, we described the bioactive compounds which plants naturally produce that can be used for industrial purposes. However, plants can also be considered as green biofactories for the production of recombinant products and recombinant proteins such as antibodies, vaccines, enzymes and growth factors. In recent years, biotechnological industries for the large-scale production of pharmaceuticals have mostly exploited *Escherichia coli*, yeast and mammalian cell cultures as production platforms. This choice was mainly due to the well-defined processes which were established by good manufacturing practices (GMPs) [106]. Since the 1980s, engineered plants have been subject of increasing interest as low-input production platforms for diagnostic reagents and pharmaceutical proteins. This “green” approach attracted attention among industries and the term plant molecular farming (PMF) was introduced into the scientific community [106,107,108]. Low expense, rapidity, scalability, flexibility, versatility, low or no-pathogen load, the robustness of the system and the possibility to manipulate the protein glycosylation pattern in plants are the major factors that led the PMF to success among several biotechnological strategies for molecule production (Figure 2) [106,107,108]. The recombinant products and proteins produced in plants are also free of both human pathogens and endotoxins, major concerns of producing recombinant proteins in mammalian cell culture and bacterial production systems [108].

Many pharmaceuticals have been produced in plants, some of which have also been tested in clinical trials [106,107]. Examples include the animal-derived peptides with therapeutic properties such as human insulin, human serum albumin and even HIV-neutralizing antibodies that have been produced from tobacco and corn seeds in the clinical trials financed by the projects Pharma-Planta and Future-Pharma [108]. Another example is a chimeric secretory IgA/G developed in transgenic tobacco plants that was marketed as a medical device (CaroRx) to prevent dental caries [107].

**Figure 3:**
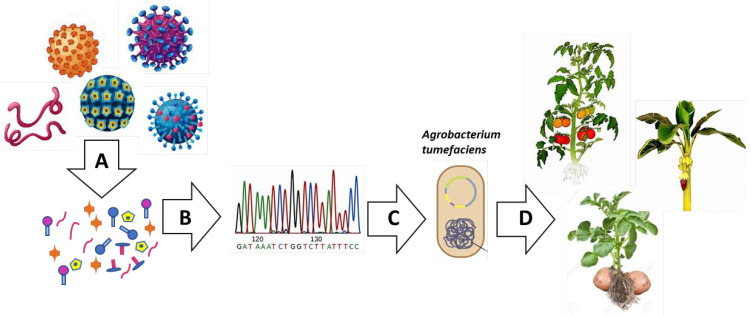
General workflow of antigen production: (**A**) Peptides tested to boost immunity response can be isolated from human and animal pathogens. In (**B**), the corresponding encoding sequences of the antigen can be amplified from the pathogen genome and cloned in an appropriate binary vector; (**C**) The binary vector carrying the antigen encoding sequence is transformed into *A. tumefaciens*; (**D**) The recombinant *A. tumefaciens* is then used to infect plants and then introduce the recombinant sequence encoding the antigen into plant cells. Finally, obtained transgenic plants become platforms for the antigen production. The use of transgenic crops (such as, tomato, banana or potato) may allow us to exploit the edible part as direct oral antigen delivery for vaccination.

Different methods are now available and used for PMF. Plant transient expression systems based on agroinfiltration or virus-based vectors that offer rapid and high-level protein expression in less than a week (Figure 3) have been developed [109]. These systems hold tremendous potential because they can be rapidly scaled up to produce proteins, emergency vaccines or biologics to meet sudden and unpredicted demand [107]. This was impressively demonstrated during the Zaire Ebola outbreak in West Africa in 2014 [106,110], as Mapp Biopharmaceuticals Inc. (San Diego, USA) produced an experimental drug (ZMapp), an anti-Ebola antibody cocktail of three neutralizing antibodies manufactured in *N. benthamiana* plants, which was approved for compassionate use due to its life-saving potential and lack of alternatives [106,107]. Today more than ever, the transient expression system may meet the demand for the high-level production of viral antigens or antiviral proteins that are needed as diagnostic reagents, vaccine candidates and even antiviral drugs to fight against the pandemic caused by the coronavirus SARS-CoV-2 [106,107]. Different plant species have been used for the stable and transient expression of recombinant proteins in either the nucleus or chloroplasts. The preferred plant species for PMF belong to the Nicotiana genus, including *N. benthamiana* and *N. tabacum*, since these are no-food/no-feed and therefore carry a reduced risk of transgenic material contaminating food and feed supplies. However, edible cereal crops, fruits and vegetables (e.g., rice, maize, tomato) are also considered suitable biofactories, considering the high yield of recombinant proteins that they can produce, the possibility of producing edible vaccines and since recombinant proteins can be targeted to accumulate in the seed. In this case, however, it is important to consider the possible competition for food production. Finally, aquatic plants, moss and algae are being used for molecular pharming [106,107]. In addition, recombinant proteins have been expressed in different plant organs, including leaves, fruit and seeds.

For example, the production of recombinant proteins in the oil bodies of plant seeds represents a viable strategy since it is a scalable, safe and cost-effective method, and the oil bodies can easily be separated from the seeds by simple centrifugation. However, this production platform is not suited for every protein and recombinant proteins must be fused with a hydrophobic component to be expressed.

Regarding this last system, one paper was recently published [111] that describes the expression of the recombinant protein human epidermal growth factor (hEGF) fused to oleosin in the seeds of transgenic *A. thaliana* plants [77]. hEGF is used in medicine and cosmetics since it plays an important role in promoting wound and ulcer healing. The authors demonstrated the efficient expression of the recombinant protein oleosin–hEGF–hEGF in oil bodies. Moreover, they demonstrated that the transgenic oil bodies had an ideal diameter to favour efficient transdermal absorption and were able to stimulate NIH/3T3 cell proliferation activity and activate the expression of EGFR in the skin [111].

An “evergreen” method for PMF is the use of the individual suspension of plant cells. This system shows similarity with bacterial and mammalian cell cultures and could be easily adjusted, with a few changes, to existing GMP regulations that require that manufacturers take proactive steps to ensure that their products are safe, pure, and effective and to minimize or eliminate instances of contamination, mix-ups and errors [106,109]. The first product obtained through plant cell cultures was approved in 2006 by the United States Department of Agriculture and it was a tobacco cell-based veterinary vaccine against Newcastle disease virus (NDV) developed by Dow Agroscience (Zionsville, IN, USA) [109]. Another successful product obtained through this in vitro culture was “Elelyso”, a recombinant form of the human enzyme glucocerbrosidase, produced and commercialized by Protalix Biotherapeutics, for the treatment of Gaucher’s disease [106].

In addition to peptides, plant cell suspension cultures are a viable technology to produce plant specialized metabolites. Pacliaxtel is among the most famous metabolites, which can be produced by cells derived from *Taxus brevifolia*. Pacliaxtel, usually sold under its trade name Taxol, is widely used to treat a number of different types of cancers such as ovarian cancer, oesophageal cancer, breast cancer, lung cancer, Kaposi sarcoma, cervical cancer and pancreatic cancer [108]. One issue related to this system could be the low final yields of the specialized metabolites that could, however, be obtained, as already stated in this review, through the elicitation of plant suspension cultures using both natural and synthetic molecules. These include plant hormones and metal salts such as salicylic acid, methyl-jasmonate, AgNO_3_, and molecules derived from fungi and bacteria [112]. For example, various cell cultures including *C. roseus*, *C. avellane*, *V. vinifera* and *S. lycopersicum*, have been shown to change their metabolic profiles when elicited with methyl jasmonate and β-cyclodextrins, used either alone or in combination [112]. In the paper by Gad et al., 2020 [27], the authors described the development of a new protocol to increase the production of hepatoprotective compounds from callus derived from the cotyledons of *Silybum marianum* L., a plant commonly known for the treatment of liver and gallbladder disorder, through the optimized supplementation of the Murashige and Skoog (MS) medium with the growth regulators 2,4-D-benzylaminopurine, myoinositol and asparagine. The results reveal that media optimization supported the synthesis of chlorogenic acid and 3,5-O-dicaffeoylquinic acid in the callus, which have anti-genotoxic activity that suppressed the mutagenic action of 4-nitro-o-phenylenediamine in *Salmonella thyphimurium* TA98.

We can foresee that a higher number of products obtained through plant biofactories will finally enter the market. Indeed, considering the high number of evolving techniques in PMF supported by novel biotechnological progresses, which are making the methods for producing biomolecules from plants more efficient, industries are investing more and more in this production platform. One example is that of the novel plant breeding techniques (NPBTs), such as the CRISPR–Cas9 system, which can be used, for example, to produce plants with no final transgenic material and that are indistinguishable from those bred by conventional breeding approaches [113].

## 5. Novel Perspectives in the Production of Plant-Derived Compounds

### 5.1. Genome Editing

The ever-increasing demand for plant-derived compounds as a food resource, medicines and/or as a building block for industrial processes has required the acceleration of the development of innovative systems for the production of useful compounds. In this context, gene/genome editing represents an innovative technology for the accurate and targeted modifications of plant traits to improve plant fitness and productivity. Genome editing takes advantage of genetic engineering-based techniques but offers faster and more accurate results. The main advantage of this technique is that it can precisely modify or add single or several nucleotides into the plant genome [109,114,115,116]. The efficacy of this novel methodology relies on the fact that the modification of a given sequence is targeted and punctual, and that no randomized positional effects can occur. One of the most popular technologies used to facilitate genome editing in plants is the clustered regularly interspaced short palindromic repeats (CRISPR)–CRISPR-associated protein 9 (Cas9). In CRISPR/Cas9 technology, the high specificity of DNA cleavage by nuclease (Cas9) is granted by an appropriated designed guide RNA (gRNA), which drives nucleases to cut the correct sequences. A main bottleneck is represented by the necessity to have a deep knowledge of the genome to be modified, in order to prevent any possible off-target effects. Today, very helpful computational off-target prediction algorithms are available and included in the databases of plant genomes. These databases rely on the presence of a comprehensive genomic dataset which allows us to quantify the possible off-target effects of gRNAs on DNA and thus select/design the best guides. Most of the genome editing strategies in plants have been addressed in the aim of the modification of agronomical interesting traits, but its use for increasing the yields of specialized metabolites of nutraceutical or pharmaceutical interest is growing. Alkaloids from Opium poppy, *Papaver somniferum*, remain elective drugs for pain relief formulations and several approaches have been pursued to manipulate their production [62,117]. Singh et al., 2019 [118], used genome editing strategies to understand the role of 4′OMT in benzyl isochinoline alkaloids (BIAs) production in opium poppy. Namely, the authors, by knocking out the 4′*omt2* gene, obtained edited plants with a reduced biosynthesis of several benzylisoquinoline alkaloids, such as S-reticuline and laudanosine, along with a considerable decrease in thebaine, codeine, noscapine and papaverine levels in a tissue-specific manner. This study proved that genome editing is an effective method to understand the regulatory role of biosynthetic genes within a pathway, thus opening the way for accurate metabolite flux manipulation. A smarter CRISPR–Cas9 strategy is the target activation-induced cytidine deaminase (Target-AID) base-editing technology, where the CRISPR-Cas9 system fused with activation-induced cytidine deaminase (AID) can be used to substitute a cytidine with a thymine, thus creating new alleles, introducing variability in several commercial crops and possibly new elite varieties. This technology allows modifying multiple genes controlling a specific metabolic pathway. For example, in tomato, this strategy has been used to increase carotenoid and especially lycopene production by modifying *Slddb1*, *Sldet1* and *Slcyc-b* genes [119]. Similar results can be obtained by using the multiplex CRISPR/Cas9 technologies where multiple SgRNA guides and nucleases are expressed at once. For example, it has been used to increase γ-aminobutyric acid (GABA) content in tomato [120]. In tomato plants, GABA is produced in larger amounts during fruit development to provide correct fruit growth and ripening [121]. Nevertheless, GABA function during vegetative and reproductive growth is not fully understood in plants. Instead, in humans, GABA is known as an inhibitory neurotransmitter [122], and reduced levels of GABA have been associated with insomnia and depressive state. In this regard, GABA enrichment in tomato fruits might contribute to provide extra GABA uptake through the diet. The authors, by using this strategy, first succeeded in targeting more genes, obtaining the simultaneous editing of four genes in tomato leaves and this resulted in a 19-fold increased production of GABA compared to control plants. Nevertheless, the negative effects on vegetative growth and reproduction were detected in increased GABA lines and further studies are necessary to understand the involvement of GABA hyperaccumulation with abnormal tomato fruit and leaf phenotypes. In all cases, these few examples suggest that genome editing represents a valuable possibility to modify crucial biosynthetic pathways, either for obtaining valuable molecules or gain novel knowledge on gene functions and regulatory mechanisms in plants.

### 5.2. Synthetic Biology

The elucidation of plant specialized metabolites biosynthesis has enabled notable advances in metabolic engineering approaches, as previously reported, but has also promoted the emergence of synthetic biology strategies. The technology relies on the reconstruction and optimization of a plant metabolic pathway in a microbial host organism. In the last ten years, synthetic biology has become one of the most valuable alternatives to native plants for the high yield production of complex and valuable compounds in a heterologous system. Microbial engineering offers several advantages, namely the fast growth of the host microorganism, which has the capability to satisfy the commercial demand; the use of fermenters, which allows scalability and the controlled production of a given molecules; along with yield in high amount and purity, without the production of side compounds. All these aspects allow us to reduce time consuming and costly processes for the production and extraction of a natural product from the native plant. Specimens within the bacteria and fungi kingdom, notably *Escherichia coli* and *Saccharomyces cerevisiae*, respectively, are the most used hosts. Once one has begun planning a synthetic metabolic strategy, an immediate decision to take is whether yeast or bacteria is the best suited system to produce a specific plant-derived metabolite. These microbial representatives have several favourable features and disadvantages. In detail, *E coli* is easy to grow and to genetically manipulate and allows the large-scale production of recombinant proteins in a short amount of time. This requires scalable and low-cost culture conditions, but it presents several disadvantages among which there is the difficulty of expressing some eukaryotic proteins, the accumulation of proteins as inclusion bodies, the degradation of expressed proteins because of host protease contamination, and also endotoxin accumulation. *S. cerevisiae* is an economic eukaryotic expression system which combines the advantages of both prokaryotic and eukaryotic expression systems. This system is mostly used to express either secretory or intracellular proteins, because it grants correct protein folding. This yeast system allows high protein yield, lesser expression time, post translational modifications and requires simple media. This system can be optimized for high level protein expression using fermenters. A major drawback consists of the hyperglycosylation of proteins, since it can provide both N- and O-linked oligosaccharides on proteins [123,124]. In summary, *S. cerevisiae* offers the opportunity to grant an easy integration of plant genomic portion through homologous recombination, and as a eukaryote organism, it contains organelles and enzymes often required for catalysing a complex plant reaction; moreover, it enables proper protein folding. Nevertheless, in many cases, *E. coli* can offer better catalytic capabilities than *S. cerevisiae*. For example, Taxol precursors have been obtained more efficiently by engineering *E coli* than *S. cerevisiae* [125]. In addition, isoprene- and isoprene-derived metabolites such as lycopene, artemisinic acid and carotenoids have been produced with a high yield in this organism, since the upstream isoprene metabolic pathway is native of *E. coli* [126]. The production of hyoscyamine and scopolamine tropane alkaloids was attained with a high titre in *S. cerevisiae* cultures, by combining the overexpression of an endogenous yeast pathway and the heterologous expression of a plant/bacterial pathway [127]. Taking into account the enzymatic requirements for a biosynthetic pathway and the different peculiarities of the hosts’ microbial organism, it is possible to modulate enzymatic efficiencies by mixing distinct strains together. Indeed, the production of complex plant compounds, especially of those highly required by the pharmaceutical market, was achieved by culturing polycultures in a single bioreactor, and thus allowing a better functional diversification of the intermediates and final product yield, without pushing metabolic flux on a single microbial line [128]. Consortium microbial strain can be useful not only for producing useful and complex intermediates or valuable final molecules, but also to metabolize starting materials to produce in a sustainable and economic manner, products of industrial interest. In this regard, several interesting papers have been published which give an overview of the actual state of commercial production of biopharmaceuticals and valuable molecules of commercial interest [123,129,130,131]. For example, bioethanol production can be recovered from agricultural waste, such as lignocellulosic biomass, by using simultaneous saccharification and co-fermentation in engineered *E. coli* [132].

In all the synthetic biology strategies, omics databases and computational tools for pathway simulations have been developed to offer the possibility of using promiscuous enzymatic capabilities for maximizing the yields of desired molecules [129,133]. However, a major hurdle in microbial platforms was the incomplete knowledge of a biosynthetic pathway leading to plant specialized metabolites. This aspect is a strong limit to the complete synthesis of the desired product and in this case, plants, such as tobacco, can be engineered to host heterologous plant enzymes, providing better results due to the compatibilities between the DNA of the source and of the host. On the other side, in addition to the higher speed of metabolite synthesis, synthetic biology can be addressed towards protein engineering. As an example, chimeric recombinant proteins could allow designing novel metabolic decorations on a plant molecule backbone, tailored for better bioactivities. Synthetic biology can lead to unpredictable challenges that are highly promising for the development of sustainable solutions for pharmaceutical, industrial and ecological processes at research and industrial scales.

## 6. Conclusions

Plants represent solutions for pharmaceutical, nutraceutical, cosmeceutical and food industries that are interested in using natural molecules with important bioactive properties. However, the over-exploitation of these resources runs the risk of reducing biodiversity, and even the extinction of important herbals accumulating important molecules. The use of in vitro culture has always represented a smart alternative for phytochemical production. The use of innovative tissue culture methods or in planta strategies is still a current methodology to turn plants and plant cells into small bio factories for the industrial production of phytochemicals, as at least observed in works published in the papers reviewed herein. In the last ten years, we witnessed incredible progress in functional genomics and metabolomics along with novel transformation strategies, e.g., genome-editing methods and synthetic biology approaches, among others. We strongly believe that these molecular innovations will preserve biodiversity, while possibly impacting the production of drugs and nutraceuticals by using plants. All these advances, in addition to contributing to meeting the social, economic and commercial demands, are based on the awareness to preserve plants as irreplaceable natural resources.

## Figures and Tables

**Table 1:** Examples of published patents that use plants for the production of biomolecules.

Patent Number	Molecule Targeted	Methodology Used or Proposed	Inventors	Assignee/Applicant
US7754943B2	Several plant molecules	Method for culturing in bioreactor C4 transgenic grasses	Brumbley et al., 2010 [39]	BSES Limited, Indooroopilly (AU)
US8338143B2	Taxol	Elicitors to enhance production in cell culture of taxus species	Bringi et al., 2012 [40]	Phyton Holdings, LLC, SanAntonio, TX (US)
US8586722B2	Starch/sugars	Antisense silencing technology to reduce amount of reducing sugars in tubers	Kossmann et al., 2013 [41]	Bayer Cropscience AG, Monheim (DE)
US8802925B2	Flavonoids	Recombinant DNA technology in Solanaceae	Luo et al., 2014 [42]	Norfolk Plant Sciences Limited, Norfolk (GB)
US10533182B2	Polyunsaturated fatty acids	Recombinant DNA technology in Brassica	Cirpus et al., 2019 [43]	BASF Plant Science GmbH, Ludwigshafen (DE)
US10743567B2	Steviol glycosides	Engineered and synthetic biology technology for production in host cells	Philippe et al., 2020 [44]	MANUS BIO INC., Cambridge, MA (US)
WO2020222233A1	Cannabinoids	Plant cell culture	Yanay and Raviv, 2020 [45]	Pluristem LTD., Haifa (IL)
US9796980B2	Isoprenoid pathway	Synthetic biology technology for production in *E. coli*	Ajikumar et al., 2017 [46]	Massachusetts Institute of Technology Cambridge, Cambridge, MA (US); National University of Singapore, Singapore (SG)
US10638679B2	Several plant-derived compounds	Cell pack cultivation	Rademacher, 2020 [47]	Fraunhofer-Gesellschaftzur Förderungderangewandten Forschung e.V., München (DE)
US8158857B2	Human protein	Human protein	Huang et al., 2012 [48]	Ventria Bioscience, Sacramento, CA (US)

**Table 2:** Commercial production of plant-derived compounds via plant cell culture modified from Krasteva et al., 2021, and Wilson et al., 2012 [87,90].

Secondary Metabolites	Species	Industrial Manufacturer	Use/Notes
Anthocyanins	*Euphorbia milii, Aralia cordata*	Nippon Paint Co., Ltd. (Japan)	Textile dyes, colouring agents for beverages
Arbutin	*Catharanthus roseus*	Mitsui Chemicals, Inc. (Japan)	Pigment, antiseptic
Betacyanins	*β vulgaris*	Nippon Shinyaku Co., Ltd. (Japan)	Food colorant and dye
Carthamin	*Carthamus tinctorius*	Kibun Foods, Inc. (Japan)	Food colorant and dye
Geraniol	*Geraminea *spp.**	Mitsui Chemicals, Inc. (Japan)	Essential oil
Ginseng	*Panax ginseng, Wild ginseng*	Nitto Denko Corporation (Japan)	Dietary supplement, cosmetics
Sanguinarin	*Papaverum sonniferum*	Vipont research laboratories (Canada)	Cosmetic anti-inflammatory
Glabridin	*Glycyrrhiza glabra*	Lonza (Switzerland)	Skin brightening
Rosmarinic chlorogenic caffeic acid	*Symphytum officinale *L.,* Saponaria Pumila*	Mibelle AG Biochemistry (Switzerland)	Cosmetics, skin care products
Phenols and flavonoids	*Gossypium herbaceum *L.**	Vytrus Biotech (Spain)	Cosmetics,
Berberines	*Coptis*	Mitsui Chemicals, Inc. (Japan)	Anti-cancer, antibiotic, anti-inflammatory
Echinacea polysaccharides	*Echinacea purpurea, Echinacea angustifolia*	Diversa (Germany)	Immunostimulant, anti-inflammatory
Paclitaxel	*Taxus *spp.**	Phyton Biotech, Inc. (USA/Germany), Samyang, Genex (Korea)	Anti-cancer
Podophyllotoxin	*Podophyllum *spp.**	Nippon Oil (Japan)	Anti-cancer
Rosmarinic acid	*Coleus blumei*	A. Nattermann & Cie. GmbH (Germany)	Anti-inflammatory
Scopolamine	*Duboisia *spp.**	Sumitomo Chemical Co., Ltd. (Japan)	Anticholinergic, anti-muscarinic
Shikonin	*Lithospermum erythrorhizon*	Mitsui Chemicals, Inc. (Japan)	Pigment, antibiotic
Echinacoside	*Echinacea angustifolia*	Institute of Biotechnological research (IRB) by Sederma (Italy)	Food supplement ingredient
Verbascoside	*Lippia citriodora*	Active Botanicals Research (ABR) (Italy)	Food supplement ingredient
Vanillin	*Vanilla planifolia*	Esca genetics corporation (USA)	Food ingredients, flavour

**Table 3:** Promising elicitation strategies used for improvement of plant-derived compounds in plant cell and organ cultures modified from [91,92,93,94,95,96,97,98,99,100,101,102,103].

Bioactive Compounds	Plant	Tissue Type	Elicitors	References
Rosmarinic acid, chicoric acid, anthocyanins	*Purple basil* *Ocimum basilicum *L. var.* purpurascen*	Callus cultures	Melatonin; and UV-C irradiation	[91]
Glycyrrhizin	*Glycyrrhiza glabra*	Hairy root cultures	Polyethylene glycol CdCl2, cellulase, mannan	[92]
Ephedrine	*Ephedra alata*	Suspension cultures	*Aspergillus niger* and yeast extract	[93]
Caffeic acid; cynaroside	*Vitex agnus castus*	Agitated shoot cultures	L-phenylalanine	[94]
Lignans	*Linum ussitatsimum *L.**	Cell suspension cultures	Salicylic acid (SA)	[95]
Aloe-emodin	*Cassia tora*	Root cultures	Chitosan; yeast extract	[96]
Thymol; p-cymene	*Carum copticum *L.**	Callus cultures	Salt stress and chitosan	[97]
Paclitaxel	*Taxus canadensis*	Plant	Chitosan-derived oligosaccharides	[98]
Tanshinone	*Salvia miltiorrhiza, S. officinalis, S. castanea*	Plant	MeJA	[99,100]
GABA	*Sesamum indicum*	Plant	Salt stress	[101]
Vinblastine, vincristine	*Catharanthus roseus*	Plant	Salt stress	[102]
Steviol glycosides	*Stevia rebaudiana*	Plant	PEG	[103]
